# A novel method for segmenting growth of cells in sheared endothelial culture reveals the secretion of an anti-inflammatory mediator

**DOI:** 10.1186/s13036-018-0107-6

**Published:** 2018-08-14

**Authors:** Mean Ghim, Kuin T. Pang, Mehwish Arshad, Xiaomeng Wang, Peter D. Weinberg

**Affiliations:** 1 Department of Bioengineering, Imperial College London, London, SW7 2AZ UK; 2 Institute of Molecular and Cell Biology, Agency for Science, Technology and Research (A*STAR), 61 Biopolis Drive, Singapore, 138673 Republic of Singapore; 3 Lee Kong Chian School of Medicine, Nanyang Technological University Singapore, 59 Nanyang Drive, Singapore, 636921 Republic of Singapore; 4 Institute of Ophthalmology, University College London, EC1V 9EL, London, UK; 5 Singapore Eye Research Institute, The Academia, 20 College Road Discovery Tower Level 6, Singapore, 169856 Republic of Singapore

## Abstract

**Background:**

Effects of shear stress on endothelium are important for the normal physiology of blood vessels and are implicated in the pathogenesis of atherosclerosis. They have been extensively studied in vitro. In one paradigm, endothelial cells are cultured in devices that produce spatially varying shear stress profiles, and the local profile is compared with the properties of cells at the same position. A flaw in this class of experiments is that cells exposed to a certain shear profile in one location may release mediators into the medium that alter the behaviour of cells at another location, experiencing different shear, thus obscuring or corrupting the true relation between shear and cell properties.

**Methods:**

Surface coating methods were developed for attaching cells only to some areas of culture-ware and preventing them from spreading into other regions even during prolonged culture.

**Results:**

Segmenting the growth of cells had no effect on cell shape, alignment and number per unit area compared to culturing cells in the whole well, but there were differences in tumour-necrosis-factor-α (TNF-α)-induced expression of vascular cell adhesion molecule-1 (VCAM-1) and intercellular adhesion molecule-1 (ICAM-1), and monocyte adherence to the monolayer.

**Conclusions:**

The results are consistent with the release of a mediator from cells exposed to high-magnitude uniaxial shear stress that has anti-inflammatory effects on activated endothelium; the mediator may be of importance in atherogenesis. Hence the new methods revealed an important property that would not have been observed without growth segmentation, suggesting that they could find more widespread application.

## Background

Vascular endothelial cells (EC) sense the shear stress generated by the flow of blood over them and respond by altering their stiffness and morphology, regulating their junctions with neighbouring cells and releasing soluble mediators [1–3], amongst other changes. Such responses, when coupled with the variation in wall shear stress (WSS) that occurs from site to site within vessels, can explain local differences in endothelial properties and may also account for the patchy development of diseases such as atherosclerosis [4–7]. Their physiological and pathological importance has stimulated many studies of the effects of shear on EC in culture.

Commonly employed devices for shearing cells include the cone-and-plate viscometer and the parallel-plate flow chamber; both allow cells to be exposed to steady, oscillatory and pulsatile flow [8, 9], but the flow is uniaxial and spatially uniform. A number of methods have been developed to overcome these restrictions, including flow chambers with tapered or branching geometries, or with a backwards facing step [10]. An alternative is to culture EC in standard petri dishes or culture plates on an orbital shaker; the orbital motion leads to variation in patterns of shear from the centre to the edge of the dish or well. The technique additionally permits high throughput and chronic exposure to flow, allowing the study of a wide range of EC responses. For example, it has enabled the demonstration that chronic and acute shear stress have opposite effects on endothelial permeability to macromolecules [11].

These more complex methods require the use of computational fluid dynamics (CFD) to characterise the spatial variation in flow. In the swirling-well system, for example, CFD has been used to show that cells at the centre of the well experience multidirectional shear stress whilst cells closer to the edge experience a nearly uniaxial shear stress [12, 13]. (The capacity to generate multidirectional shear stress is important as recent studies have suggested that it correlates with lesion formation *in vivo* [14, 15].) Taking advantage of the spatial variation in shear stress requires spatially-resolved assays of cellular responses. At the simplest level, this can mean harvesting cells from different regions prior to analysis. Higher-resolution methods involve the use of optical – particularly microscopical – assays, such as those involving colorimetric or fluorimetric readouts.

In both cases, the properties of cells in one region are assumed to be related to the shear stresses occurring in the same region, allowing comparisons to be made between sites. However, there is a potential flaw in these methods: EC release soluble mediators and microparticles, a process that – as noted above – can depend on flow characteristics [16–18]. The mediators will affect cells in regions other than the one where they were released, due either to general mixing in the swirling medium on an orbital shaker or to advection from one location to another in parallel-plate devices. This may corrupt or hide true effects of shear on cell behaviour. We have speculated that an effect of this type accounts for the apparently identical influence of different shear profiles on transcellular transport of large particles [12].

In some previous studies [19], cells have been seeded only in specific regions of the device. This theoretically avoids the flaw descried above: if cells are restricted to one area, corresponding to one shear profile, the properties of those cells cannot be influenced by mediators from cells exposed to other shear profiles, and they cannot influence such cells. We show below, however, that in practice cells seeded in one location spread to other regions during chronic experiments.

Here we describe and evaluate methods for promoting cell adhesion in some regions of devices whilst using surface passivation to prevent growth elsewhere, even after many days. Using the swirling well device as a model, we demonstrate an effect of shear on cell behaviour that is hidden if cells are allowed to grow over the entire well rather than being restricted to specific segments of it. More specifically, we demonstrate that endothelium releases anti-inflammatory mediators when exposed to certain patterns of shear, a finding that may be important for understanding atherogenesis. The demonstration that important shear responses are revealed only when using the new methods mandates their wider use.

## Methods

### Cell isolation and culture

Human Umbilical Vein Endothelial Cells (HUVEC) were isolated by applying the protocol of Jaffe et al. [19] with minor modification to cords obtained from donors with uncomplicated labour at the Hammersmith Hospital, UK. The cells were cultured in flasks coated with 0.1% gelatin using Endothelial Growth Medium (EGM-2) containing the EGM-2 supplement kit (Lonza, Switzerland). Passages 3–5 were used for experiments.

Cells of the human acute monocytic leukemia suspension line (THP-1) were obtained from the American Type Culture Collection (USA). They were maintained in RPMI 1640 supplemented with 10% FBS, 2 mM L-glutamine, 100 U/mL penicillin and 100 μg/mL streptomycin (Sigma-Aldrich, UK). Passages 6–20 were used for experiments.

All cells were maintained at 37 °C in a humidified incubator under 95% air/5% CO_2_.

### Region-specific coating and culture of HUVEC

To coat specific areas of 6-well plates with substrate or passivator, PDMS rings were fabricated from a master mould that was designed using competer aided design software (Solidworks 2016) and printed with an Ultimaker 2+ 3-D printer using polylactic acid filaments. A mixture of PDMS base and curing agent (90.9% base and 9.1% curing agent) was poured directly into the mould, degassed and cured at 80 °C for 1 h before removal.

**Fig. 1:**
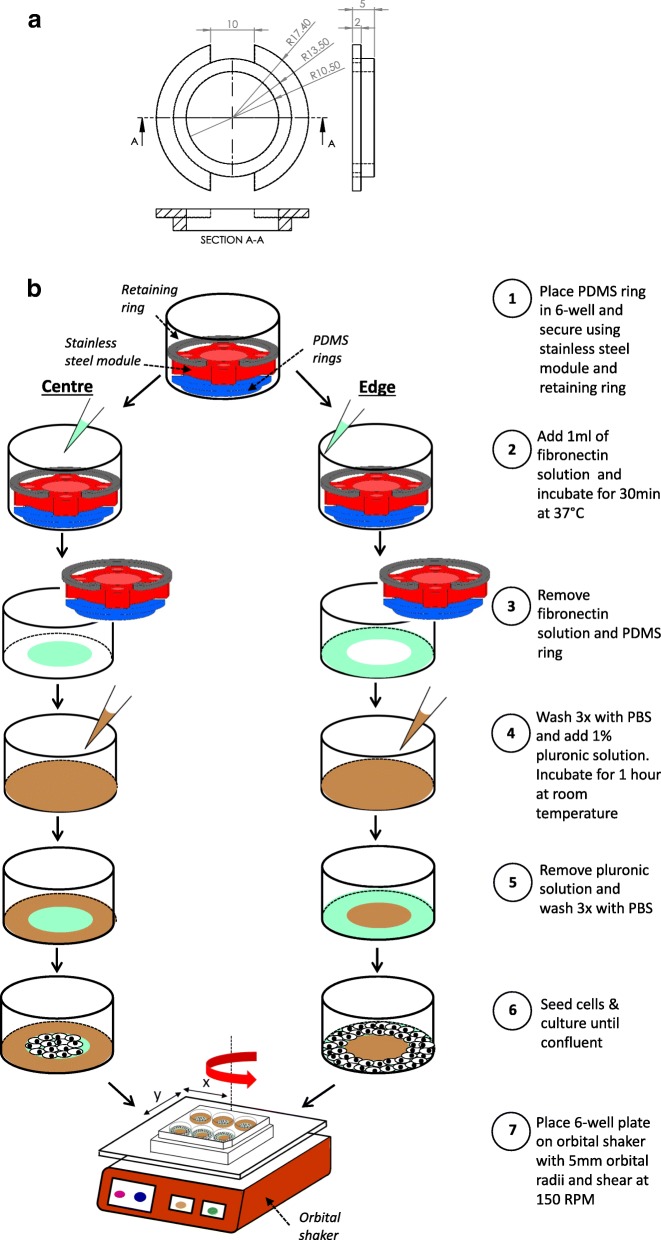
**a** Dimensions (in mm) of the PDMS ring used to segment the wells. **b** Schematic of the methods for allowing cell growth only in the centre or edge of a 6-well plate

A PDMS ring (Fig. 1a) was placed in each well and secured using a custom-made stainless steel module and retaining ring (Misumi, UK) to give a tight seal between the bottom of the PDMS ring and the base of the well. One ml of a 5 μg/ml fibronectin solution was pipetted into the centre or the edge of the well and left for 30 min at 37 °C. The fibronectin solution and then the PDMS ring were removed from the well, which was washed three times with phosphate-buffered saline (PBS). The non-fibronectin coated region was passivated by the addition of a 1% Pluronic F-127 solution (Sigma-Aldrich, UK) to the well for 1 h. The Pluronic solution was removed and the wells were washed three times with PBS. The coating procedure is illustrated in Fig. 1b. Note that it only works with non-tissue culture treated wells. Wells coated only with fibronectin, without the PDMS ring, allowed cells to grow over the entire base of the well and were used as a control.

HUVEC were seeded at 1.5 × 10^5^ cells/well, and medium was replaced after 24 h to remove the non-adhered cells. They were cultured for another 2 days until confluent.

### Application of shear stress and TNF-α

Once the HUVEC were confluent, the medium in each well was replaced with 1.9 mL of fresh medium and the 6-well plate was placed on the horizontal platform of an orbital shaker (POS-300, Grant Instruments) in the incubator for 3 days. The platform orbited in the horizontal plane with an orbital radius of 5 mm and angular velocity of 150 rpm.

To expose HUVEC to Tumor Necrosis Factor-α (TNF-α), 1.9 μl of a 10 μg/mL solution was added to the medium in each well after 48 h of shear, and the cells were then sheared for another 24 h before assays were performed.

### Counting HUVEC

HUVEC were stained with Hoechst 33342 (2 μg/mL; Thermofisher Scientific, USA) in the incubator for 30 min, prior to fixation with 4% paraformaldehyde (PFA) for 10 min. Ten fields were imaged at each location of interest using an inverted SP105F fluorescence microscope (Brunel Microscopes, UK) with a 20× objective, 365/50 nm excitation filter, 400 nm dichroic mirror and 460/50 nm emission filter. The number of HUVEC was quantified using a custom script written in MATLAB (The MathWorks) which distinguished stained nuclei by intensity and area thresholding.

To quantify the number of HUVEC as a function of distance along a radius, the well was washed 3 times with PBS and cell nuclei were stained with DRAQ5 at a 1:1000 dilution for 15 min. A tile scan was performed from the edge to the centre of the well using an inverted TCS SP5 confocal microscope (Leica, UK) with a 10× objective. DRAQ5 was excited at 633 nm and detected at 670-750 nm. Nuclei were identified and counted as described above.

### THP-1 adhesion assay

THP-1 cells in suspension culture were centrifuged at 200 x *g* for 5 min and resuspended in RPMI 1640 medium at 1 × 10^6^ cells/ml. One μl of a 1 mg/ml solution of Calcein-AM (Life Technologies, USA) was added to 1 mL of the cell suspension and incubated for 30 min in the incubator. Cells were centrifuged at 200 x *g*, resuspended in EGM-2, applied to the monolayers of HUVEC in 6-well plates at a density of 1 million per well and left for 1 h without swirling. Non-adhered THP-1 cells were removed with three washes of pre-warmed medium and cells were fixed with 4% PFA for 15 min. Ten fields were imaged at each location of interest using the inverted SP105F fluorescence microscope with a 20× objective, 470/40 nm excitation filter, 495 nm dichroic mirror and 525/50 nm emission filter. The number of adhered THP-1 cells was quantified using a custom MATLAB script, as above.

### Cell orientation and shape index

Following 3 days of shear, cells were fixed with 4% PFA for 10 min. The wells were washed 3 times with PBS and nuclei were stained with DRAQ5 and imaged from the edge to the centre with the confocal microscope, as above.

The images were post processed using ImageJ and a custom MATLAB script. A maximum projection image was created from the 43 slices in each z-stack. Area and intensity thresholding were used to distinguish nuclei from background. Individual images in the tile scan were stitched together using the image stitching plugin in ImageJ [20] and then subdivided into 1 mm intervals along the radius. Ellipses were fitted to the nuclei and the orientation of each nucleus was calculated as the angle between the long axis of the fitted ellipse and a vector running from the centre of the well to the centroid of the ellipse (y-axis), with positive numbers indicating that the end of the nucleus nearest the center of the well was displaced to the right of the reference vector.

The shape index (SI) of each nucleus was defined as [21]:

$$ SI=4\pi A/{P}^2 $$

where A is the area and P is the perimeter of the ellipse. An SI of 1 indicates a circle whereas a value of 0 indicates a line.

### Immunofluorescence staining of ZO-1

After exposure to shear, HUVEC were fixed with 4% PFA for 10 min, permeabilised with 0.1% Triton X-100 (Sigma-Aldrich, UK) for 5 min and blocked with 1% bovine serum albumin (BSA) for 1 h at room temperature. Cells were then incubated with rabbit anti-human zonula occludens-1 (ZO-1) (#13663, Cell Signaling Technology, USA) at a 1:200 dilution in 1% BSA overnight at 4 °C, followed by three washes in PBS and incubation with Alexa Fluor 488-labelled goat anti-rabbit IgG (A11008, ThermoFisher Scientific, USA) at a 1:300 dilution in PBS at room temperature for 1 h. Nuclei were stained with DRAQ5 as above. Secondary antibody was imaged with the inverted Leica TCS SP5 using a 10× objective, 488 nm excitation and detection at 505-535 nm. DRAQ5 was imaged using the wavelengths described above.

### SDS-PAGE and western blotting

HUVEC were lysed using radioimmunoprecipitation assay buffer (Sigma-Aldrich, UK) supplemented with Halt protease and phosphatase inhibitor (ThermoFisher Scientific, USA). Extracted proteins were separated by sodium dodecyl sulfate polyacrylamide gel electrophoresis (SDS-PAGE). Gels were transferred onto a PVDF membrane (Merck Millipore, USA). Blots were probed with rabbit anti-human VCAM-1 (sc-8304, Santa Cruz Biotechnology, USA), rabbit anti-human ICAM-1 (sc-7891, Santa Cruz Biotechnology, USA), or mouse anti-human calnexin (LS-C179860, Source BioScience, UK), followed by goat anti-rabbit (ab6721, Abcam, UK) or goat anti-mouse (sc-2005, Santa Cruz Biotechnology, USA) horseradish peroxidase-conjugated secondary antibodies. Blots were incubated using Clarity ECL substrate (Biorad, USA) before the bands were imaged (Biospectrum imaging system, UVP, UK). Densitometry was performed using Image Studio Lite software (LI-COR, USA).

### Computational fluid dynamics

Flow simulations were carried out with Star CCM+ (version 11.02.009, CD-Adapco, USA). A single well of a 6-well plate was represented as a cylinder of height of 10 mm and radius of 17.4 mm. The geometry was discretized using a structured cylindrical mesh with 360,000 grid elements. The explicit unsteady model was used. A no-slip condition was imposed at all walls and surface tension was neglected. The top surface of the cylinder was defined as a pressure outlet. The dynamic viscosity and density of medium were 0.78 × 10^3^ Pa.s and 1003 kg/m^3^ respectively.

The rotation of the well was modelled by introducing a translating gravitational force with the form

$$ \left[\mathrm{x},\mathrm{y},\mathrm{z}\right]:\left[{\mathrm{A}\upomega}^2\cos \left(\upomega \mathrm{t}\right),{\mathrm{A}\upomega}^2\sin \left(\upomega \mathrm{t}\right),-9.81\right] $$

where A is the orbital radius of the shaker, ω is the angular velocity and *t* is time.

The Volume of Fluid model was used to track the free surface of the liquid, which had a height of 2 mm when the well was stationary. Time steps of 1 × 10^− 4^ s were each iterated 5 times. Maximum WSS at the base of the well was used to assess convergence. A mesh independence study was performed using 720,000 grid elements, and no difference was observed.

### Computation of shear metrics

Post-processing employed MATLAB to obtain the shear stress acting on the monolayer, here termed wall shear stress (WSS) by analogy with the shear exerted on endothelium in vivo. The instantaneous WSS vectors (${\overrightarrow{\tau}}_w$) from one complete revolution were used to calculate time-average WSS (TAWSS), the Oscillatory Shear Index (OSI) [22], and transverse WSS (transWSS) [22], reflecting, respectively, the temporal average of the instantaneous vectors, the tendency of the instantaneous vectors to deviate from the direction of the mean vector, and the average of the components of the instantaneous vectors acting perpendicular to the mean vector:

$$ TAWSS=\frac{1}{T}{\int}_0^T\left|{\overrightarrow{\tau}}_w\right| dt, $$ where $$ \left|{\overrightarrow{\tau}}_w\right|\equiv \sqrt{{\tau_x}^2+{\tau_y}^2+{\tau_z}^2} $$

$$ OSI=\frac{1}{2}\left(1-\frac{\left|{\int}_0^T{\overrightarrow{\tau}}_w dt\right|}{\int_0^T\left|{\overrightarrow{\tau}}_w\right| dt}\right) $$

$$ \mathrm{TransWSS}=\frac{1}{T}{\int}_0^T\left|{\overrightarrow{\tau}}_w\bullet \left(\overrightarrow{n}\times \frac{{\overrightarrow{\tau}}_{mean}}{\left|{\overrightarrow{\tau}}_{mean}\right|}\right)\right| dt $$

### Statistical analysis

Data are presented as mean ± 1 standard error of the mean. Statistical analyses were performed by one-way or two-way ANOVA using GraphPad Prism 6 (GraphPAD Software Inc.,USA). The criterion for significance was *p* < 0.05.

## Results

### Flow simulations

**Fig. 2:**
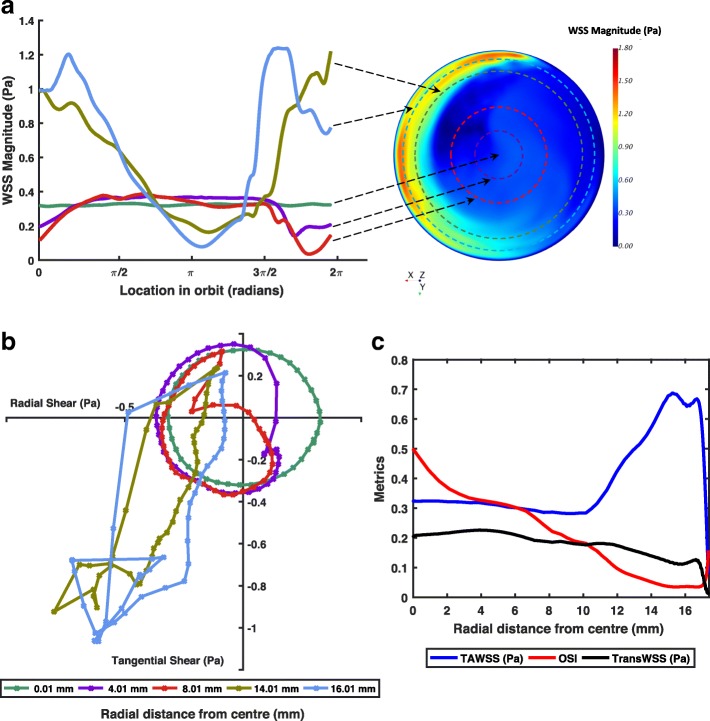
**a** Map of the instantaneous WSS magnitude acting on the base of a 6-well plate on the orbital shaker, and plot of WSS magnitude at different radial distances from the centre throughout one cycle (2π radians). Arrows indicate the radial position for each curve on the WSS magnitude map. **b** Polar plot of the magnitude and direction of instantaneous WSS vectors during one cycle. Each curve applies to one radial distance from the centre of the well. The curve represents the path travelled by the tip of the WSS vector, with its origin at 0,0. Points on the curves are spaced at 10 ms intervals. **c** Plots of three WSS metrics – TAWSS, OSI, and transWSS – with respect to radial distance

The movement of the platform induced the medium to swirl. Figure 2a shows a snapshot of the shear produced on the base of the well. The pattern rotated around the well in synchrony with the orbit of the platform. At the centre of the well, the magnitude of the shear stress vectors was approximately constant during each orbit, but the direction of the vector rotated around the well at a constant rate, resulting in a perfectly multidirectional shear stress profile, as expected from considerations of radial symmetry. At the edges of the well, radial components of the vector were suppressed, particularly components in the positive (outwards) direction, resulting in a nearly uniaxial shear stress profile, but the direction of the instantaneous vectors along this axis and their magnitude fluctuated during each orbit (Fig. 2b). On average, magnitudes were higher at the edge than in the centre. In this respect, the solution differs from that previously obtained for 12-well plates on the same platform [12], leading to low magnitude multidirectional flow (LMMF) towards the centre and high magnitude uniaxial flow (HMUF) towards the edge.

### Region-specific culture of HUVEC

The choice of dimensions for the regions was based on results from the flow simulations. To expose cells only to LMMF, fibronectin was applied to a circular region having the same centre as the well itself and a radius of 10.5 mm. For exposure only to HMUF, the fibronectin-coated region was an annulus with the same centre, an inner radius of 13.5 mm and an outer boundary at the wall of the well (radius 17.4 mm). Figure 2c shows that TAWSS was relatively low (around 0.3 Pa) from the centre to a radial distance of 10.5 mm, and that from a radial distance of 13.5 mm to the outer wall, TAWSS was everywhere above 0.5 Pa except for a narrow region affected by the no-slip condition at the wall. Figures 2b and c show that OSI and transWSS – two indices of multidirectional shear stress – were higher in the central disc than in the outer annulus. The areas of the two regions differed by less than 10%.

**Fig. 3:**
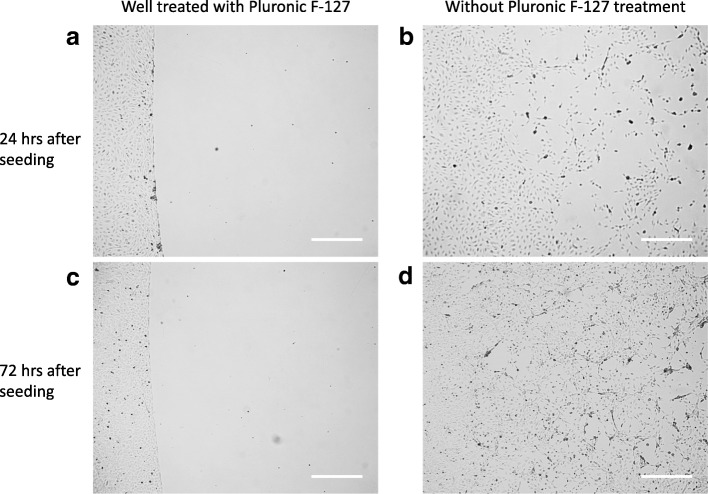
Microscope images showing that Pluronic F-127 prevents HUVEC adhesion to the region without fibronectin coating**.** No HUVEC were attached to the part of the well surface that had not been pre-treated with fibronectin, prior to passivation with Pluronic F-127, after 24 h (**a**) and 72 h (**c**) of growth. Without Pluronic F-127 passivation, HUVEC were attached to the surface without fibronectin 24 h after seeding (**b**) and had proliferated further by 72 h (**d**). (Scale bar = 500 μm)

Pluronic passivation abrogated adhesion of cells to the region not coated with fibronectin; growth was restricted to the fibronectin coated region even after 6 days of culture. If the passivation step was omitted, cells seeded – albeit sparsely – and spread to the regions not coated with fibronectin and proliferated there (Fig. 3).

### Morphology, orientation and number of HUVEC in segmented wells and full wells

**Fig. 4:**
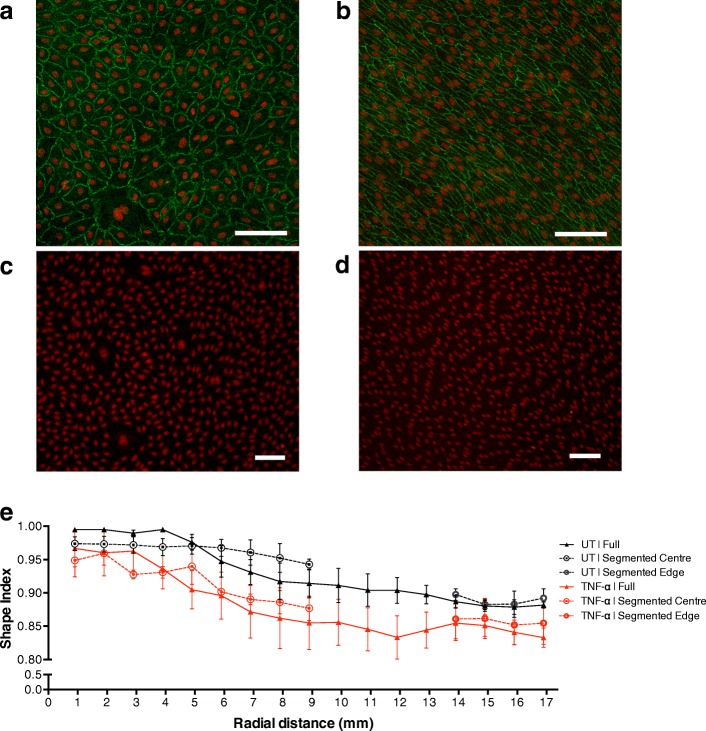
Nuclear (red) stain shows the morphology of sheared HUVEC (**a**) in the centre and (**b**) at the edge of a full well, and (**c**) in the centre and (**d**) at the edge of a segmented well (scale bar = 100 μm). **a** and **b** also show cell outlines, delineated by immunostaining of ZO-1. Note the alignment and elongation of cells at the edge but not at the centre, and the lack of difference between full and segmented wells. **e** No significant difference in nuclear Shape Index, indicating roundness, between HUVEC grown in full wells and segmented wells was seen for untreated or TNF-α treated HUVEC. Cells were more elongated near the edge of the well. A tendency for greater elongation in TNF-α-treated HUVEC was not consistently significant across locations

HUVEC in the annulus were visibly aligned and elongated, while HUVEC at the centre of the well exhibited a cobble stone morphology and no alignment (Fig. 4a–b).

The roundness of nuclei was quantified as their Shape Index; it decreased with distance along the radius, demonstrating that the nuclei become more elongated under HMUF (Fig. 4e). At each radial location there was no statistically significant difference in the Shape Index for HUVEC grown in segmented wells and full wells. The same result was obtained after TNF-α treatment. The treatment itself did have a tendency to increase elongation, but this trend was inconsistent, reaching statistical significance at only a few radial locations.

**Fig. 5:**
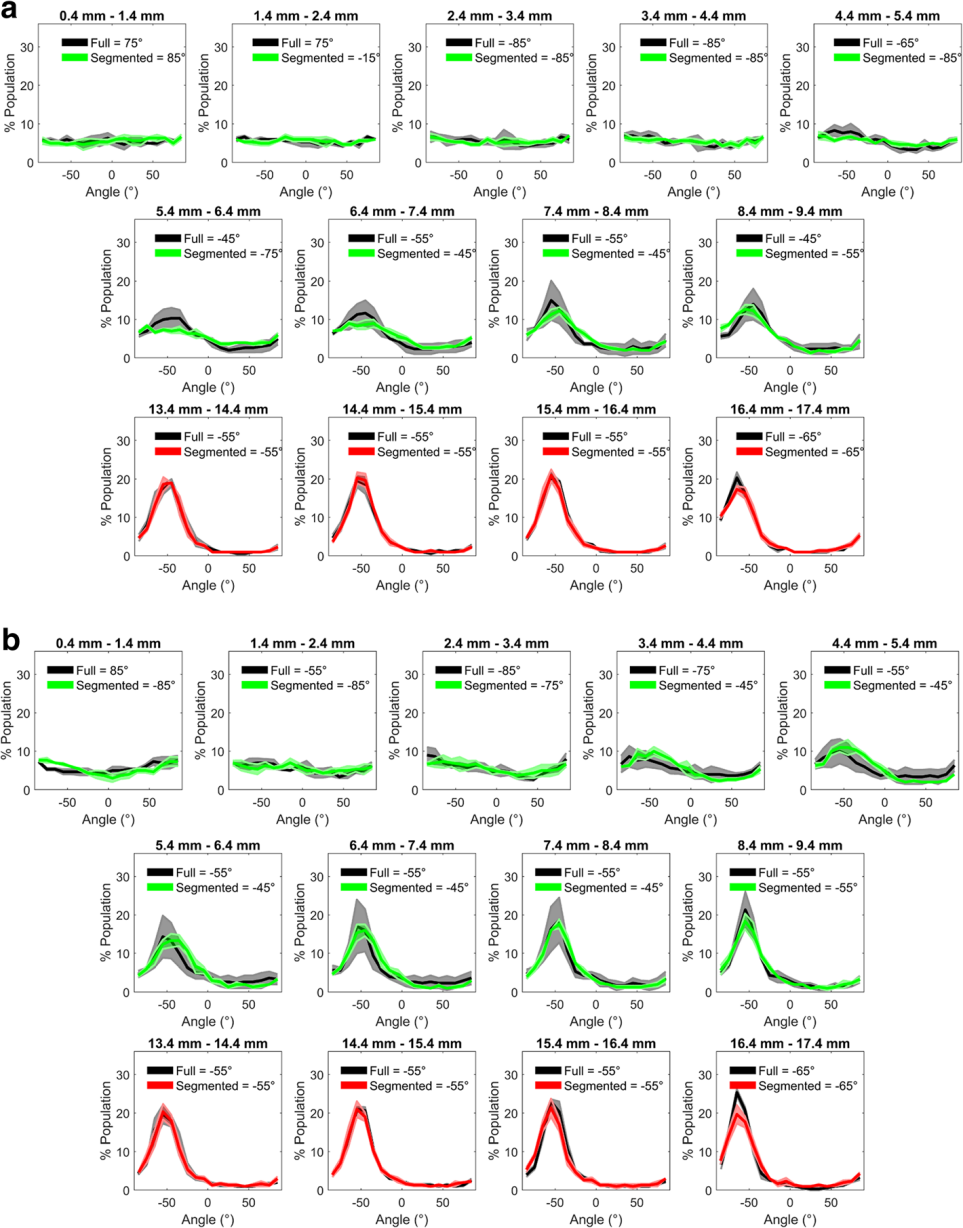
Orientation of HUVEC nuclei in wells where the cells were growing only in the central region (green), only near the edge (red) or everywhere (grey). Each panel shows a different radial distance from the centre of the well. Mean data are presented as lines of best fit (obtained using seventh-order polynomials) and shaded areas show the standard error of the mean for 3 independent experiments. **a** shows results for untreated HUVEC and (**b**) shows results for TNF-α treated HUVEC. The modal orientation, with and without segmentation, is shown at the top of each panel. panel. Note the absence of alignment near the well centre

Similarly, at each radial location and for both untreated and TNF-α treated HUVEC, there was no statistically significant difference in alignment between cells grown in a segmented well compared to a full well (Fig. 5). There was again a small effect of TNF-α at some radial locations and, for unknown reasons, at radial distances between approximately 4 mm and 9 mm there was a larger standard error in the alignment of HUVEC grown in a full well compared to those grown in segmented well.

**Fig. 6:**
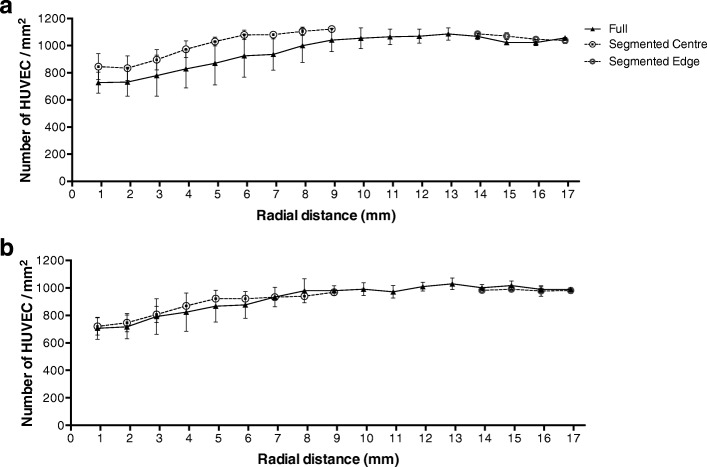
No significant difference was observed between full and segmented wells in the number density of (**a**) untreated and (**b**) TNF-α-treated HUVEC at different radial locations. In both cases, there were more cells per unit area at the edge than the centre of the well. (Two-way ANOVA and Bonferroni’s post hoc test; *n* = 3)

The number of HUVEC per mm^2^ increased with radial distance under all conditions. Once more, there was no statistically difference between HUVEC grown in segmented wells and full wells, with or without TNF-α treatment, and no consistent significant effect of TNF-α (Fig. 6a-b).

### Monocyte adhesion and adhesion molecule expression in segmented and full wells

**Fig. 7:**
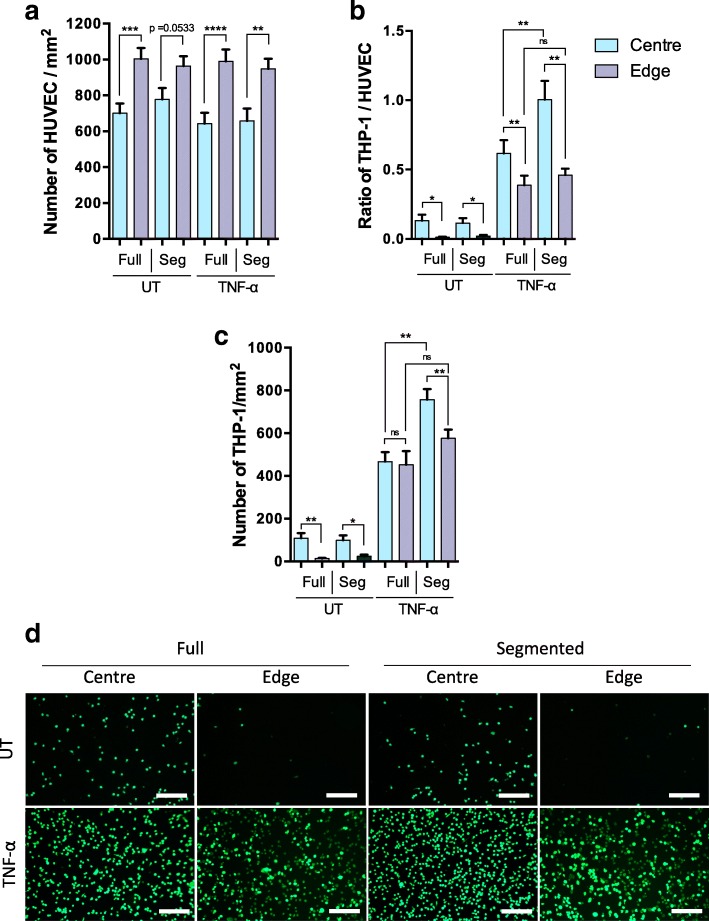
Monocyte adhesion on HUVEC grown in full wells or only at the centre or edge of segmented wells. **a** The density of HUVEC, **b** The ratio of THP-1 monocytes to HUVEC, **c** the number of THP-1 and (**d**) representative images of Calcein-AM-stained THP-1 adhered on HUVEC (scale bar = 200 μm), all shown for the centre and edge of full or segmented wells, with and without TNF-α treatment. (UT = untreated; one-way ANOVA followed by Fisher’s Least Significant Difference test, *n* = 6; * *p* < 0.05, ** *p* < 0.01, *** *p* < 0.001, and **** *p* < 0.0001)

The number of adhered THP-1 monocytes in each region was normalised by the number of HUVEC in the same region (Fig. 7a).

There was a significantly higher THP-1:HUVEC ratio in the centre of the well than at the edge in both untreated and TNF-α treated HUVEC (Fig. 7b). A significantly higher ratio was also observed in TNF- α treated than in untreated monolayers for both regions of the well, whether segmented or not.

In the absence of TNF-α, there was no significant effect of segmentation on the adhered THP-1:HUVEC ratio in either the centre or the edge of the well. However, segmentation of wells containing TNF-α treated HUVEC resulted in a significant (*p* < 0.01) increase in the monocyte adhesion ratio at the centre but not at the edge of the well. Note that since segmentation had no effect on HUVEC density in either region (Figs. 6a-b and 7a), this result cannot be explained by the use of normalisation. Furthermore, the same result was obtained for the absolute density of THP-1 (Fig. 7c).

**Fig. 8:**
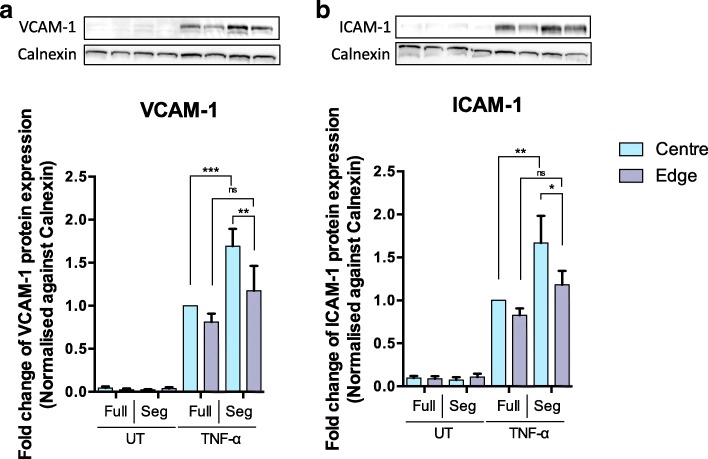
Western blots of (**a**) VCAM-1 and (**b**) ICAM-1 expression by HUVEC grown in full wells or only at the centre or edge of segmented wells, with and without TNF-α treatment. Calnexin was used as a loading control. (UT = untreated; one-way ANOVA followed by Fisher’s Least Significant Difference test, *n* = 5; * p < 0.05, ** p < 0.01, *** p < 0.001)

Protein expression of VCAM-1 and ICAM-1 by HUVEC, detected using Western Blotting (Fig. 8a-b), was consistent with the monocyte adhesion data: segmentation of wells containing TNF-α treated HUVEC resulted in a significant (p < 0.01) increase in the expression of both molecules at the centre but not at the edge of the well.

## Discussion

This swirling-well system allows cells to be exposed to a range of physiological shear stress profiles depending on the location of the cells within the well. For example, cells at the centre of the well experience multidirectional shear stress whilst cells nearer to the edge experience pulsatile uniaxial shear stress resembling that seen in straight, unbranched arterial segments *in vivo* [12, 13]. The capacity to generate multidirectional shear stress is important because recent studies have suggested anatomical co-localisation of transWSS with a high predilection for lesion formation [14, 15].

Use of the swirling-well system has increased in recent years with many studies using it to examine effects of multidirectional flow and uniaxial flow on cultured endothelium concurrently [23–27]. However, endothelial cells are known to release soluble or microparticulate mediators and this may also vary depending on the flow characteristics [16–18]. Such mediators will be mixed by the swirling medium and affect cells everywhere in the well, leading to decoupling between the site at which a particular shear stress profile is exerted and the sites at which its effects can be observed. The same also applies to other culture systems, even those with uniform flow properties [28].

To overcome this issue, the swirling-well system was modified here to permit growth of cells only in specific regions of the well. If cells grow only at the centre, for example, they cannot be affected by mediators released at the edge of the well in response to uniaxial shear stresses. Cells typically adhere more avidly to hydrophilic surfaces than to hydrophobic ones. For this reason, multi-well cell culture plates, formed from hydrophobic polystyrene, are pre-treated by plasma oxidation. Alternatively, hydrophobic surfaces can be coated with extracellular matrix proteins such as fibronectin. In the absence of such surface modification, cells adhesion is drastically reduced but not abrogated. In the present study, hydrophobic 6-well culture plates were coated with fibronectin at the edge or centre of the well to promote cell adhesion, and the non-fibronectin coated regions were passivated with Pluronic F-127 to completely prevent it. The method is simple to use but effective: passivation successfully restricted cells to the fibronectin coated regions even during prolonged culture.

The method was developed to test the theoretical prediction that mediators released by endothelial cells in a shear-dependent fashion can have effects on cells at other locations in the culture apparatus, and to provide a means for preventing such effects. HUVEC confined to the edge or the centre of the well exhibited the same elongation, alignment and density as HUVEC at the same radial locations in wells where cells were permitted to grow everywhere. The same phenomenon was observed when the HUVEC had been exposed to TNF-α as well as to shear (although there were minor effects of the TNF-α itself). Hence there was no evidence that these three properties were affected by the shear-induced release and mixing of mediators.

Shear stress profiles influence vascular inflammation by modifying endothelial gene expression [29, 30]. Activation of endothelial cells and subsequent expression of proteins such as E-selectin, VCAM-1, and ICAM-1 promote the recruitment, arrest, and transmigration of circulating monocytes [31]. These steps are critical to the inflammatory process [32]. Expression of these markers can also be activated by cytokines such as TNF-α and Interleukin 6 [33].

In this study, we performed monocyte adhesion assays on pre-sheared monolayers treated with TNF-α or left untreated as a control. Quantification of adhered monocytes after shear but in the absence of TNF-α showed that LMMF was more pro-inflammatory than HMUF. These results agree with a similar study by Dardik et al. [19]. Our result was not affected by whether the cells were confined only to the centre or the edge of the well.

Pretreatment with TNF-α in addition to shear increased monocyte accumulation in both regions and regardless of whether cell growth was segmented, as expected. Notably, however, in the TNF-α treated HUVEC, segmentation increased monocyte adhesion at the centre of the well but had no effect at the edge. This finding was statistically significant (*p* < 0.01) and robust, being independent of whether the data were normalised by HUVEC density or not (Fig. 7b and c). Furthermore, expression of VCAM-1 and ICAM-1 followed the same trend: segmentation increased expression at the centre of the well but had no effect at the edge (Fig. 8a and b). As far as we are aware, this is the first demonstration that segmenting the growth of cells exposed to spatially-varying shear profiles can affect the properties of those cells.

Not only is the effect novel but it has allowed the deduction of an endothelial property of potential importance in vascular pathophysiology: we interpret the result to mean that when cells were cultured both at the centre and at the edge, those at the edge, exposed to HMUF, released a mediator that suppressed inflammation at the centre of the well, where cells were exposed to LMMF. Such an effect would be seen when cells are cultured across the whole well, but not when they are cultured only at the centre. No effect of growth segmentation was seen at the edge of the well, where cells would be exposed to the putative mediator not only when allowed to grow everywhere but also when allowed to grow only at the edge, where the mediator is released. The effect was not seen in the absence of TNF-α, implying that the mediator influences some process resulting from the action of this cytokine.

## Conclusions

The existence of an anti-inflammatory mediator released from cells in response to uniaxial flow clearly may be of importance in the pathogenesis of atherosclerosis. Additional work is required to determine the nature of the mediator and to understand its effects more fully. The new method itself provides a means for such investigations, since it enables accurate harvesting of the cells of interest, and allows for the simple collection of medium conditioned by cells exposed to well-defined flows. Here we are concerned to show that the postulated theoretical possibility of shear-dependent mediators corrupting apparent relations between shear and endothelial properties can indeed be observed in cell culture, and that it is therefore necessary to adopt methods, such as segmenting cell growth, that can prevent such effects.

## Abbreviations

BSA: Bovine serum albumin
CFD: Computational fluid dynamics
EC: Endothelial cells
EGM: Endothelial growth medium
HMUF: High magnitude unidirectional flow
HUVEC: Human umbilical vein endothelial cells
ICAM-1: Intercellular adhesion molecule-1
LMMF: Low magnitude multidirectional flow
OSI: Oscillatory Shear Index
PBS: Phosphate-buffered saline
PDMS: Polydimethylsiloxane
PFA: Paraformaldehyde
SI: Shape Index
TAWSS: Time averaged wall shear stress
THP-1: Human acute monocytic leukemia suspension line
TNF-α: Tumor necrosis factor-α
transWSS: Transverse wall shear stress
VCAM-1: Vascular cell adhesion molecule-1
WSS: Wall shear stress
ZO-1: Zonula occludens-1
